# Tourette syndrome and brain stimulation therapy: a systematic review and meta-analysis of current evidence

**DOI:** 10.3389/fpsyt.2025.1478503

**Published:** 2025-02-18

**Authors:** Alwaleed K. Aloufi, Jalal A. Zahhar, Mahmoud W. Bader, Maher B. Almutairi, Abdulqader Alaaldeen, Omar E. Hetta, Abdulaziz M. Gammash, Saleh Almuntashiri, Ibrahim S. Binrabaa, Ahmad Alsaleh, Moayyad AlSalem

**Affiliations:** ^1^ College of Medicine, King Saud Bin Abdulaziz University for Health Sciences, Jeddah, Saudi Arabia; ^2^ King Abdullah International Medical Research Centre, Jeddah, Saudi Arabia; ^3^ Psychiatry Section, Department of Medicine, King Abdulaziz Medical City, Ministry of the National Guard-Health Affairs, Jeddah, Saudi Arabia

**Keywords:** Tourette Syndrome, repetitive transcranial magnetic stimulation, deep brain stimulation, Yale Global Tic Severity Scale, obsessive-compulsive disorder, Yale-brown obsessive compulsive scale

## Abstract

**Background:**

Tourette syndrome (TS) is a neurological disorder characterized by tics, often associated with obsessive-compulsive disorder (OCD). Severe cases may require interventions such as deep brain stimulation (DBS) or repetitive transcranial magnetic stimulation (rTMS).

**Methods:**

A thorough search was performed across PubMed/Medline, Embase, (CENTRAL), and Google Scholar. Studies comparing DBS and rTMS efficacy for TS were included if they reported YGTSS before and after treatment. Two independent reviewers screened the search results, extracted data, and assessed study quality using standardized tools.

**Results:**

22 studies met the inclusion criteria, with a total of 222 participants. Analysis of RCTs investigating post-intervention rTMS vs baseline showed a statistically insignificant decrease in YGTSS (MD = -5.01, 95% CI: [-10.8, 0.79], P= 0.090) but a statistically significant decrease in YBOCS (MD = -6.6; 95% CI: [-11.64, -1.55], P= 0.010). However, post-intervention rTMS in RCT and non-randomized trials vs baseline showed a significant decrease in YGTSS (MD = -11.6; 95% CI: [-18.25, -4.94], P < 0.001) and YBOCS (MD = -7.5; 95% CI: [-11.85, -3.15], P < 0.001). Post-intervention DBS in RCT and non-RCTs vs baseline showed a significant decrease in YGTSS (MD = -18.29; 95% CI: [-24.93, -11.64], P < 0.001) and YBOCS (MD = -4.76; 95% CI: [-7.30, -2.21], P < 0.001). Analysis of RCTs investigating Post-intervention DBS vs baseline showed a significant decrease in YGTSS (MD = -14.71; 95% CI: [-19.78, -9.63], P <0.001) and YBOCS (MD = -5.04; 95% CI: [-8.28, -1.80], P = 0.002).

**Conclusion:**

Our analysis revealed both DBS and rTMS improved TS and OCD symptoms, however the effect of rTMS on TS in RCTs was insignificant, suggesting DBS stimulation is more effective. Despite this, clinicians may still opt for rTMS before DBS due to its less invasive nature, the limited number of high-quality RCTs, and the lack of studies directly comparing rTMS and DBS.

**Systematic Review Registration:**

https://www.crd.york.ac.uk/prospero/display_record.php?ID=CRD42023386856, identifier CRD42023386856.

## 1 Introduction

Tourette Syndrome (TS) is a neurological disorder characterized by a spectrum of motor and vocal tics - involuntary, repetitive movements or vocalizations (1). The onset of these symptoms generally surfaces between the ages of three and nine (2). The severity of the symptoms is commonly measured using the Yale Global Tic Severity Scale (YGTSS). YGTSS is a widely used assessment tool designed to measure the severity and impact of tics in individuals diagnosed with TS, as recommended by the American Academy of Neurology guideline (3). It comprises several domains, including frequency, intensity, complexity, and impairment related to both motor and vocal tics. Despite significant advancements in neuroscience, the etiology of TS remains vague, with hypotheses revolving around abnormalities in certain brain regions (including the basal ganglia, frontal lobes, and cortex), and the neurotransmitters (dopamine, serotonin, and norepinephrine) that mediate nerve cell communication (4).

The intricate pathology of TS is believed to be multifactorial, involving both genetic predispositions and potential environmental influences (5). Adding layers of complexity to the clinical manifestation and management of the condition, TS is often comorbid with other behavioral disorders such as attention deficit hyperactivity disorder (ADHD) and obsessive-compulsive disorder (OCD) (6), which is assessed using the Yale-Brown Obsessive Compulsive Scale (YBOCS). YBOCS is a standardized assessment tool utilized to measure the severity of OCD symptoms (7, 8). It evaluates the presence and intensity of obsessions and compulsions across several domains, including time spent, distress caused, and interference with daily functioning.

While there is no definitive cure for TS, a multitude of treatment strategies have been adopted to manage the symptoms and improve the quality of life for those affected. These strategies encompass cognitive-behavioral therapy (CBT), pharmacological interventions, deep brain stimulation (DBS), and repetitive transcranial magnetic stimulation (rTMS) (9, 10). Currently the management of Tourette patients consists of the following stages: first, psychological education and social support for mild conditions; second, pharmacological therapy and behavioral intervention; and third, invasive or non-invasive neuromodulation such as DBS or rTMS in severe or refractory cases (11).

Studies that compare the efficacy of DBS and rTMS in TS patients are scarce, mostly due to methodological limitations. Published studies are often conflicting (12–19), thus the development of evidence-based guidance to direct clinical decision-making in the selection of therapeutic regimen for severe or refractory TS is important. Therefore, we aimed to compare the efficacy of DBS and rTMS in TS patients. Our primary objective was to examine change in YGTSS in patients with TS before and after each intervention, and our secondary objectives were to investigate the effect of DBS on different brain areas, and the effect of brain stimulation on OCD symptoms by analyzing the change in YBOCS.

## 2 Methods

### 2.1 Study design and protocol

This systematic review and meta-analysis were conducted in accordance with the Preferred Reporting Items for Systematic Reviews and Meta-Analyses (PRISMA) guidelines. The study protocol was registered in the Prospective Register of Systematic Reviews (PROSPERO) database with registration number CRD42023386856.

### 2.2 Eligibility criteria

Inclusion criteria required that the studies report YGTSS before and after the application of any brain stimulation therapy, including rTMS and DBS, in patients with Tourette Syndrome. These patients must have been diagnosed according to the criteria set forth in the Diagnostic and Statistical Manual of Mental Disorders, the International Classification of Diseases, or the Chinese Classification of Mental Disorders. Only studies published in the English or Arabic languages were considered for inclusion. Studies were excluded if they were conducted on non-human subjects or if they did not report YGTSS. Moreover, studies were excluded if they included in their analysis patients who had initiated or changed their pharmacological treatment dosage within four weeks before receiving brain stimulation therapy.

### 2.3 Search strategy

A thorough search through multiple databases was conducted to identify relevant published papers to our study objectives. Databases searched included PubMed/Medline, Embase, Cochrane Central Register of Controlled Trials (CENTRAL), and first 10 pages of google scholar. The search strategy is comprehensively outlined in **Supplementary Table S1**.

### 2.4 Data extraction and study selection

Each article selected was independently reviewed by two authors, with any disagreements being settled either through mutual agreement or by seeking the opinion of a third author. When necessary, further details were requested from the authors of the studies to clarify eligibility criteria. Additionally, the reasons for excluding any articles from the review were documented. Corresponding authors were contacted to provide any missing data when necessary, if we did not receive a response within 1 month, data was verified from other published meta-analyses. The data that were extracted are the following: First Author Name, Year of study publication, Study design, country where the study was conducted, inclusion criteria, exclusion criteria, Sample size for each arm, range of age included in the study, mean age in each arm, gender, duration of intervention in weeks, target area(s) in the brain, frequency used/technique, mean duration of disease, Side effects reported due to intervention, baseline and post intervention YGTSS/YBOCS and standard deviation/standard error.

### 2.5 Risk of bias and quality assessment

Two independent reviewers used either the revised Cochrane risk of bias tool for randomized trials (20) or the Risk Of Bias In Non-randomized Studies - of Interventions tool (21) to assess the studies included. Any conflict was resolved by a third author.

### 2.6 Data analysis

Data analysis was conducted using Review Manager (RevMan) 5.4.1. An inverse variance random-effects model was utilized for all analyses. The threshold for statistical significance was established at P ≤ 0.05, ensuring a 95% confidence interval. Statistical heterogeneity was assessed through the I^2^ and P values derived from the chi-square test. In case the heterogeneity was more than 50%, a sensitivity analysis was conducted. A funnel plot was used to visually assess publication bias. The pooled mean difference of YGTSS and YBOCS were used to assess the change in severity of symptoms in TS and OCD patients, respectively.

## 3 Results

### 3.1 Study selection

**Figure 1:**
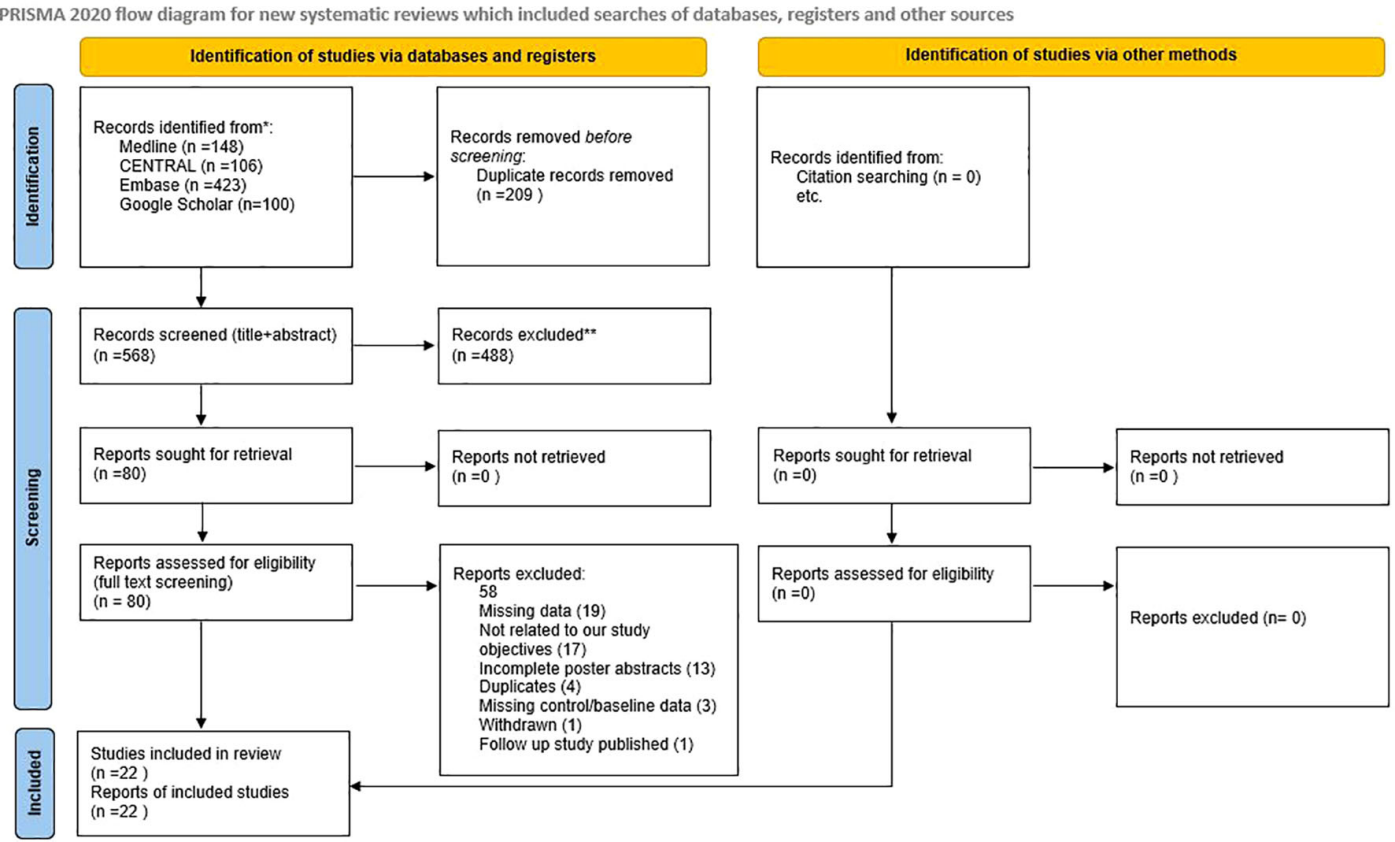
Flow chart of study selection.

We identified 777 studies, of which 209 were excluded due to duplication (**Figure 1**). A total of 568 studies were screened by title and abstract, of which 488 were considered irrelevant to the stated objective. Further, 80 studies were assessed for eligibility, and 58 studies were excluded after full-text screening. The results in Jackson N. Cagle’s study (22) reported no specific numbers; the study was excluded after receiving no response from the author. The study by Michael S. Okun (23) included patients who were part of a more recently published follow-up study by P. Justin Rossi (24), and was therefore excluded. Lastly, results in Marie-Laure Welter’s study (16) were missing, therefore, the results were selected from the last published systematic review (25) after receiving no response from the author. Finally, 22 studies were included in the review.

### 3.2 Characteristics of included studies

The 22 included studies had a total of 222 patients (**Table 1**). 14 studies were using DBS. Out of the 14 studies using DBS, 12 were randomized controlled trials (RCTs). The remaining 2 studies were non-randomized trials (non-RCTs). Of the 22 included studies, 8 were using rTMS. Of the 8 rTMS studies, 4 were RCT. The remaining 4 studies were non-RCTs The risk of bias assessment showed an overall low to medium risk for randomized studies and high risk for the non-randomized studies (**Supplementary Figures S1**, **S2**).

**Table 1:** Baseline characteristics of included studies.

Intervention	Author Name	Study Year	Country	Study Design	No. of Patients Included	Age, mean, range, yrs	Gender (male/female)	Duration of Disease, mean, yrs
DBS	Morreale et al. (12)	2021	UK	RCT	14	33.67, 18-60	14/8	26.52
DBS	Baldermann et al. (13)	2021	Germany	RCT	7	26.125, 20-32	6/2	19
DBS	Müller-Vahl et al. (26)	2021	Germany	RCT	9	29.4, 18-47	7/3	–
DBS	Cappon et al. (27)	2019	UK	RCT	11	34.3, 24-59	9/2	27.63
DBS	Welter et al. (15)	2017	France	RCT	16	30.7, 19-57	12/4	–
DBS	Haense et al. (14)	2016	Germany	RCT	10	36, 19-52	7/4	–
DBS	Rossi et al. (24)	2016	USA	RCT	4	34.4, 28-39	2/3	28.8
DBS	Schoenberg et al. (28)	2015	USA	RCT	5	28.2, 18-34	5/0	–
DBS	Kefalopoulou et al. (29)	2015	UK	RCT	15	34.7, 24-55	11/4	25.86
DBS	Huys et al. (30)	2014	Germany	Non-randomized Trial	8	33.25, 19-56	5/3	14
DBS	Cannon et al. (31)	2012	Australia	Non-randomized Trial	11	33, 18-50	8/3	22.6
DBS	Ackermans et al. (32)	2011	Netherlands	RCT	6	40.33, 35-48	6/0	33
DBS	Welter et al. (16)	2008	France	RCT	3	32, 30-36	1/2	23
DBS	Maciunas et al. (33)	2007	USA	RCT	5	28.2, 18-34	5/0	–
rTMS	Kahl et al. (18)	2021	Canada	Non-randomized Trial	10	11, 9-15	8/2	–
rTMS	Landeros-Weisenberger et al. (34)	2015	USA	RCT	20	33.3, -	16/4	28.8
rTMS	Wu et al. (35)	2014	USA	RCT	12	14.5, 10-22	9/3	8.5
rTMS	Le et al. (36)	2013	China	Non-randomized Trial	25	10.61, 7-16	22/3	3.13
rTMS	Kwon et al. (37)	2011	South Korea	Non-randomized Trial	10	9.57, 9-14	10/0	–
rTMS	Mantovani et al. (17)	2006	Italy	Non-randomized Trial	8	33.5, -	8/0	18.6
rTMS	Orth et al. (19)	2005	UK	RCT	5	29 (median), 19-52	4/1	–
rTMS	Chae et al. (38)	2004	USA	RCT	8	34.9, 19-60	5/3	28.4

### 3.3 Synthesized findings

**Figure 2:**
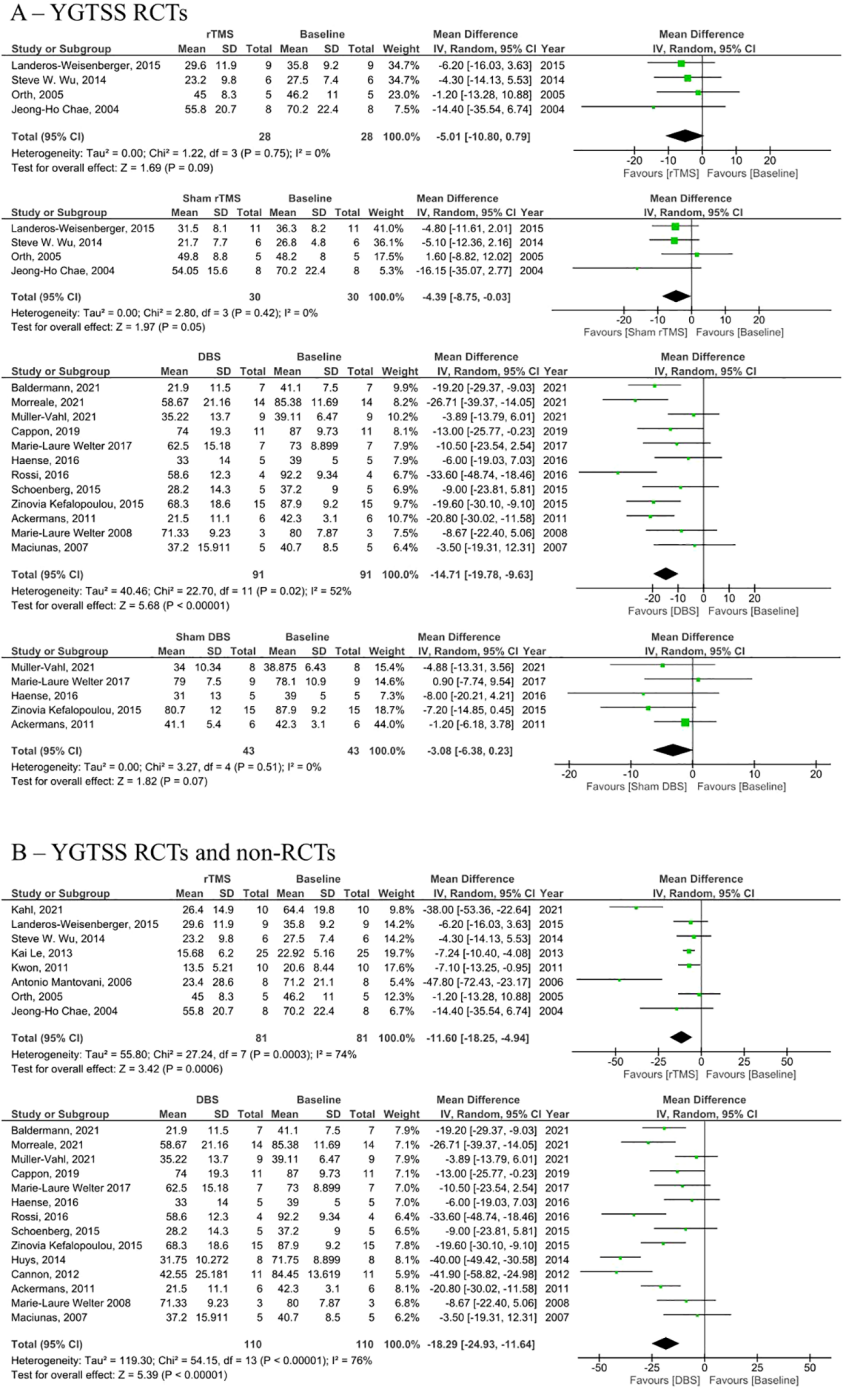
Forest plots comparing YGTSS pre and post rTMS, DBS, and sham stimulation in tourette syndrome patients. **(A)** YGTSS change in RCTs only, **(B)** Combined YGTSS change in RCTs and non-RCTs. rTMS, repetitive transcranial magnetic stimulation, DBS, deep brain stimulation, SD, standard deviation, CI, confidence interval.

**Figure 3:**
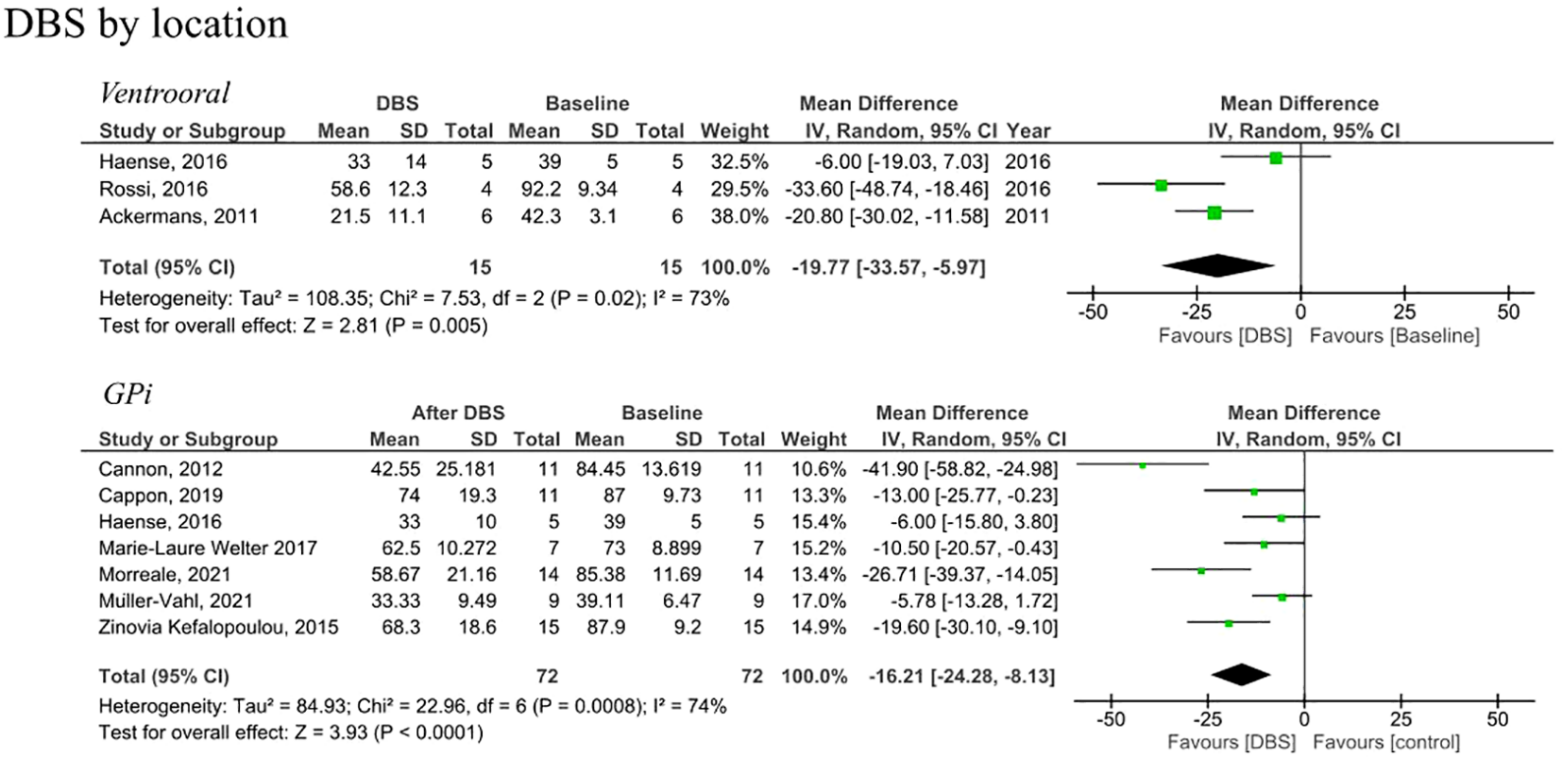
Forest plots comparing YGTSS pre and post DBS in tourette syndrome patients by anatomical location of stimulation. DBS, deep brain stimulation, Gpi, globus pallidus internus, SD, standard deviation, CI, confidence interval.

**Figure 4:**
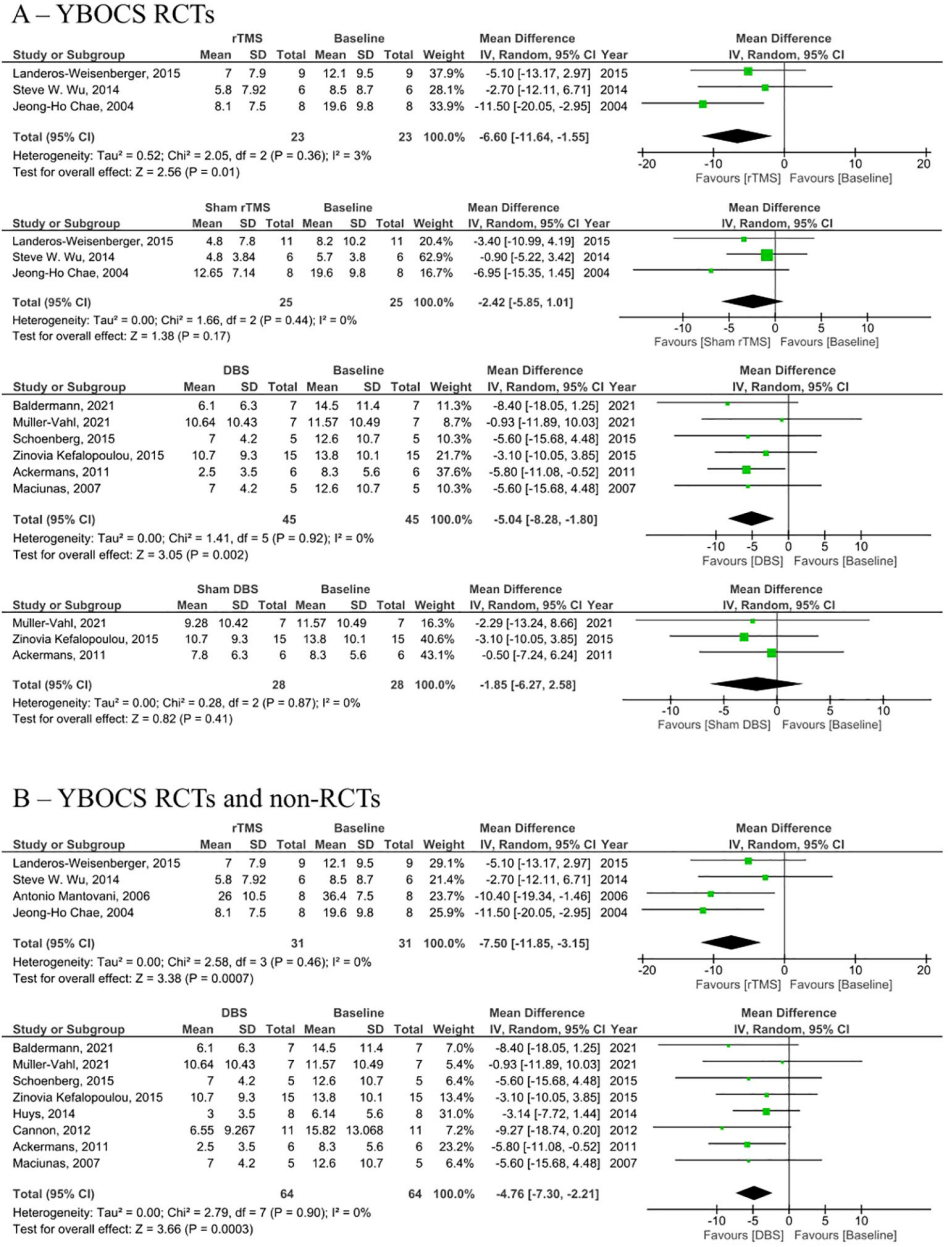
Forest plots comparing YBOCS pre and post rTMS, DBS, and sham stimulation in patients with obsessive-compulsive disorder. **(A)** YBOCS change in RCTs only, **(B)** Combined YBOCS change in RCTs and non-RCTs. rTMS, repetitive transcranial magnetic stimulation; DBS, deep brain stimulation; SD, standard deviation; CI, confidence interval. Forest plots comparing YGTSS pre and post rTMS, DBS, and sham stimulation in tourette syndrome patients.

Analysis of RCTs investigating post-intervention rTMS vs baseline showed a statistically insignificant decrease in YGTSS (MD = -5.01, 95% CI: [-10.8, 0.79], P = 0.090, I^2^ = 0%; **Figure 2A**). None of the studies showed a significant difference between the rTMS and baseline (**Figure 2A**). Further combined analysis of RCTs and non-RCTs investigating post-intervention rTMS vs baseline showed a significant decrease in YGTSS after rTMS (MD = -11.60; 95% CI: [-18.25, -4.94], P < 0.001, I^2^ = 74%; **Figure 2B**), sensitivity analysis was conducted by removing Kahl et al. (18) which reduced the heterogeneity to 51% (**Supplementary Figure S3**). Additionally, analysis of sham rTMS studies showed a marginally significant decrease in YGTSS compared to baseline (MD = -4.39; 95% CI: [-8.75, -0.03], P = 0.050, I^2^ = 0%; **Figure 2A**). Further analysis of RCTs investigating post-intervention DBS vs baseline showed a significant decrease in YGTSS following DBS (MD = -14.71; 95% CI: [-19.78, -9.63], P < 0.001, I^2^ = 52%; **Figure 2A**). Combined analysis of RCTs and non-RCTs investigating post-intervention DBS vs baseline also showed a significant decrease in YGTSS compared to baseline (MD = -18.29; 95% CI: [-24.93, -11.64], P < 0.001, I^2^ = 76%; **Figure 2B**), sensitivity analysis did not show a significant change in heterogeneity. Furthermore, analysis of sham DBS studies showed no significant difference in YGTSS compared to baseline (MD = -3.08; 95% CI: [-6.38, 0.23], P = 0.070, I^2^ = 0%; **Figure 2A**). Subgroup analysis by location of DBS showed a greater decrease in YGTSS in studies stimulating the thalamic ventrooral region vs studies stimulating the globus pallidus internus (GPi) region (MD = -19.77; 95% CI = [-33.57, -5.97], P = 0.005, vs MD = -16.21; 95% CI: [-24.28, -8.13], P < 0.001; **Figure 3**). Sensitivity analysis for the GPi studies was performed and showed a decrease in heterogeneity to I^2^ = 56% after removing Cannon et al. (**Supplementary Figure S4**). Sensitivity analysis for the thalamic ventrooral studies was performed and showed a decrease in heterogeneity to I^2^ = 50% after removing Haense et al. (**Supplementary Figure S5**). On combined analysis of RCTs and non-RCTs, the effect of rTMS on YBOCS showed a significant decrease in YBOCS after rTMS compared to baseline (MD = -7.5; 95% CI: [-11.85, -3.15], P < 0.001, I^2^ = 0%; **Figure 4B**); subsequent analysis of RCT studies exclusively also revealed a significant decrease in YBOCS after rTMS compared to baseline (MD = -6.6; 95% CI: [-11.64, -1.55], P = 0.010, I^2^ = 3%; **Figure 4A**). Furthermore, combined analysis of RCTs and non-RCTs examining the effect of DBS on YBOCS showed a significant decrease in YBOCS after DBS compared to baseline (MD = -4.76; 95% CI: [-7.30, -2.21], P < 0.001, I^2^ = 0%; **Figure 4B**); a further analysis of RCTs alone on the effect of DBS on YBOCS showed a significant decrease in YBOCS after DBS compared to baseline (MD = -5.04; 95% CI: [-8.28, -1.80], P = 0.002, I^2^ = 0%; **Figure 4A**). Additional analysis of sham rTMS and sham DBS studies showed no significant difference in YBOCS compared to baseline (**Figure 4A**). Primary and secondary outcomes showed no publication bias by visual inspection of funnel plot (**Supplementary Figures S6**–**S8**) (20, 21).

## 4 Discussion

The present study examined the therapeutic efficacy of rTMS and DBS in the treatment of TS and OCD. The results indicate that both rTMS and DBS exhibited effectiveness in improving symptoms in both diseases. Notably, rTMS demonstrated greater efficacy in improving OCD symptoms, while DBS was found to be more effective in TS, as rTMS failed to show effectiveness in the analysis of RCTs.

It is worth noting that few studies have directly compared the efficacy of DBS and rTMS, with most previous investigations primarily focusing on exploring the therapeutic potential of either intervention. Our findings also closely align with the previous meta-analysis by Xiaofeng Lin et al. (25). However, it is important to acknowledge that their study did not include an analysis of sham studies, and subsequent articles incorporating new evidence have been published since then.

Prior studies evaluating the YGTSS have yielded mixed results regarding the effectiveness of rTMS (17–19, 34–38). In our investigation, the YGTSS from RCTs showed no significant difference between the rTMS group and baseline (**Figure 2A**). However, when non-RCTs were included in the analysis, the rTMS group exhibited a significant decrease in YGTSS compared to the baseline (**Figure 2B**). This suggests a potential methodological limitation that has not been previously acknowledged in the literature. Furthermore, our analysis of sham rTMS studies demonstrated a marginally significant difference in YGTSS compared to the baseline, which suggests that the effectiveness seen in non-RCTs is possibly due to a placebo effect.

Conversely, DBS demonstrated a significant decrease in YGTSS compared to baseline, with this decrease observed in both RCTs and non-RCTs. Moreover, the analysis of sham DBS studies indicated no significant difference in YGTSS compared to the baseline, providing further evidence that the symptom reduction associated with DBS is attributable to the stimulation rather than the surgical procedure or a placebo effect. Additionally, DBS exhibited a more pronounced decrease in YGTSS in the thalamic ventrooral region compared to the Gpi, although the number of ventrooral studies that we were able to include in the analysis was limited, so this result should be considered with caution.

Furthermore, the YBOCS was assessed following rTMS and DBS interventions, revealing a significant decrease in compared to the baseline. The analysis of sham rTMS and sham DBS studies demonstrated no significant difference in YBOCS compared to the baseline, this also suggests the effectiveness of rTMS and DBS in these patients was not due to a placebo effect.

While both DBS and rTMS have demonstrated efficacy in patients with TS and OCD, it is important to consider the potential side effects associated with these interventions. In several articles, DBS was associated with more severe side effects, including infections, headaches, dizziness, infection, and an increase in tic severity, as indicated in **Supplementary Table S2**.

There are several limitations to consider in our study. Firstly, none of the included studies directly compared DBS and rTMS, which introduces a significant gap in our understanding of their relative efficacy. The lack of direct comparison is primarily attributed to the limited number of studies available on this topic. Furthermore, the heterogeneity in the frequency and location of stimulation between the studies further complicates the comparison of these interventions. Secondly, while our study includes findings from various trials, it is essential to acknowledge the limited number of high-quality randomized controlled trials (RCTs) available on this subject. The scarcity of such trials restricts the strength of evidence supporting our conclusions. Additionally, the potential for publication bias should be considered, as studies with negative or inconclusive results may be less likely to be published, potentially skewing the available evidence base. Thirdly, it is important to acknowledge that not all studies reported adverse effects associated with the therapies under investigation. This missing data regarding adverse effects introduces a potential bias and limits our comprehensive understanding of the safety profiles of DBS and rTMS. Therefore, caution should be exercised in interpreting the adverse effect data presented in our study. Lastly, the large heterogeneity observed across the included studies can be attributed to various factors. One major contributing factor is the diverse patient characteristics within the study populations. Patients differed significantly in terms of age, comorbidities, and medications used, among other factors. The lack of standardized patient selection criteria and control of confounding variables may have influenced the observed heterogeneity and introduced variability in the treatment outcomes.

It is essential to consider these limitations when interpreting the findings of our study, as they highlight areas for further investigation and underscore the need for more rigorous research to establish the comparative efficacy and safety of DBS and rTMS. Future studies should focus on directly comparing DBS and rTMS in randomized controlled trials to provide robust evidence regarding their relative efficacy and safety. Such research should aim to standardize stimulation protocols, patient selection criteria, and outcome measures to minimize heterogeneity and enhance the comparability of findings. Subgroup analysis including demographic variables should be considered in future studies as they might generate different risk ratios. Additionally, efforts should be made to report adverse effects comprehensively to establish a clearer understanding of the safety profiles of these interventions. Addressing these gaps will contribute significantly to optimizing treatment strategies for tic symptoms and obsessional symptoms.

## 5 Conclusion

Our study provides valuable insights into the management of TS and OCD, overall showing the effectiveness of rTMS and DBS in treating both diseases, especially in advanced disease. However, the analysis of rTMS effect on TS showed conflicting results in RCTs vs non-RCTs, likely due to the limited number of RCTs or a placebo effect. It is crucial to consider the limitations of the current literature and the lack of direct comparisons between DBS and rTMS. Further randomized controlled trials comparing both modalities directly are needed.

## Data Availability

The original contributions presented in the study are included in the article/**Supplementary Material**. Further inquiries can be directed to the corresponding author.
