# Supplementary material for: Tourette syndrome and brain stimulation therapy: a systematic review and meta-analysis of current evidence
Source: Front Psychiatry. 2025 Feb 18;16:1478503. doi: 10.3389/fpsyt.2025.1478503 (PMC11876145; doi:10.3389/fpsyt.2025.1478503)
Supplement: Supplementary file 1 [file Supplementaryfile1.docx]

Supplementary Material

**Supplementary Table S1. Medline search strategy**

| 1 | exp Tourette Syndrome/ |
| --- | --- |
| 2 | exp Tics/ |
| 3 | Tourette$.mp. |
| 4 | tics$.mp. |
| 5 | gille$.mp. |
| 6 | vocal tics$.mp. |
| 7 | motor tics$.mp. |
| 8 | neuropsychiatric movement$.mp. |
| 9 | exp Deep Brain Stimulation |
| 10 | exp Electric Stimulation Therapy/ |
| 11 | exp Transcranial Magnetic Stimulation/ |
| 12 | exp Transcranial Direct Current Stimulation/ |
| 13 | TMS.mp. |
| 14 | rtms$.mp. |
| 15 | DBS.mp. |
| 16 | brain stimulation.mp. |
| 17 | transcranial magnetic$.mp. |
| 18 | Theta Burst$.mp. |
| 19 | 1 or 2 or 3 or 4 or 5 or 6 or 7 or 8 |
| 20 | 9 or 10 or 11 or 12 or 13 or 14 or 15 or 16 or 17 or 18 |
| 21 | 19 or 20 |

**Supplementary figure S1.** Risk of bias assessment for randomized controlled trials


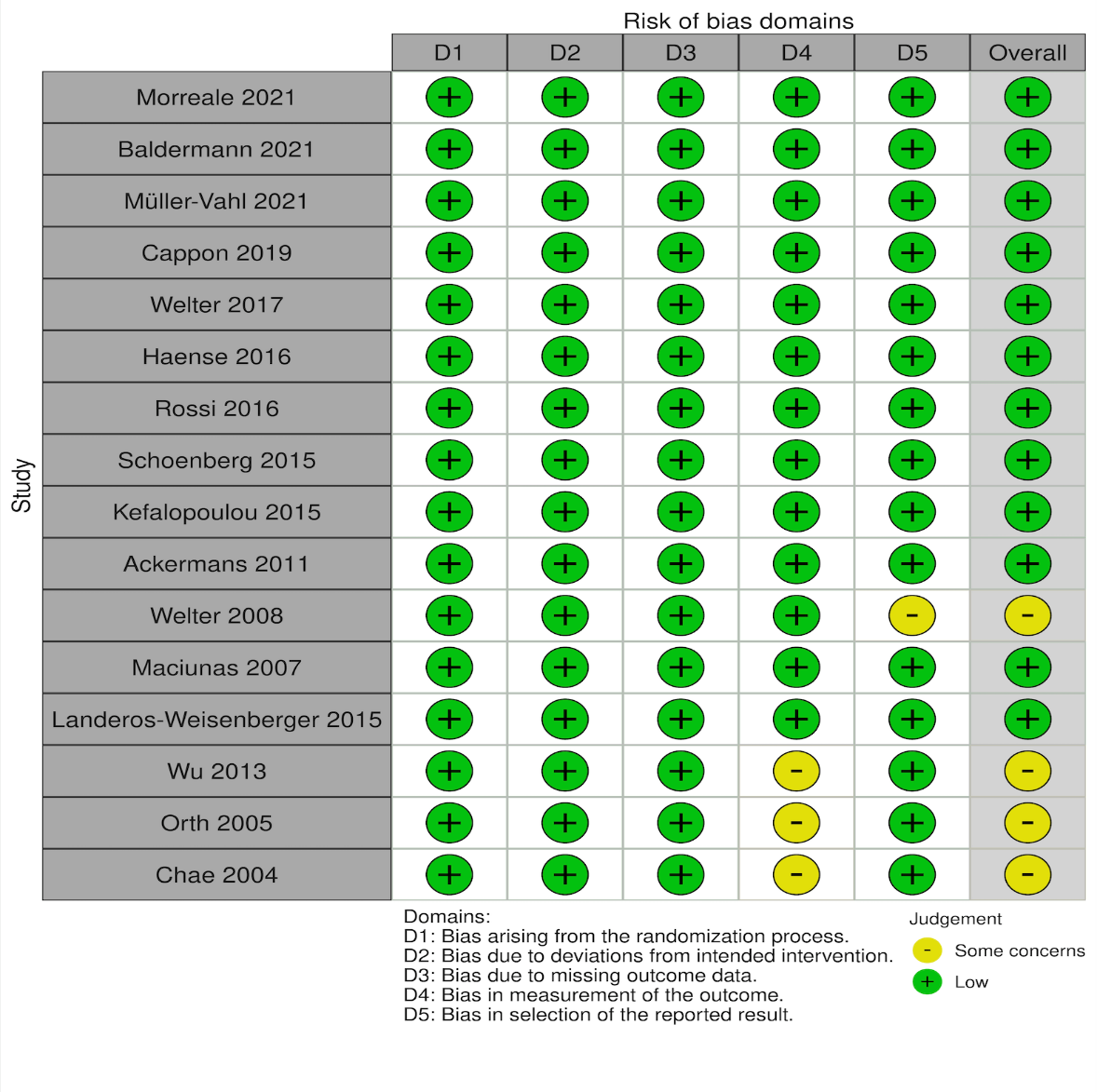


**Supplementary figure S2.** Risk of bias assessment for non-randomized controlled trials


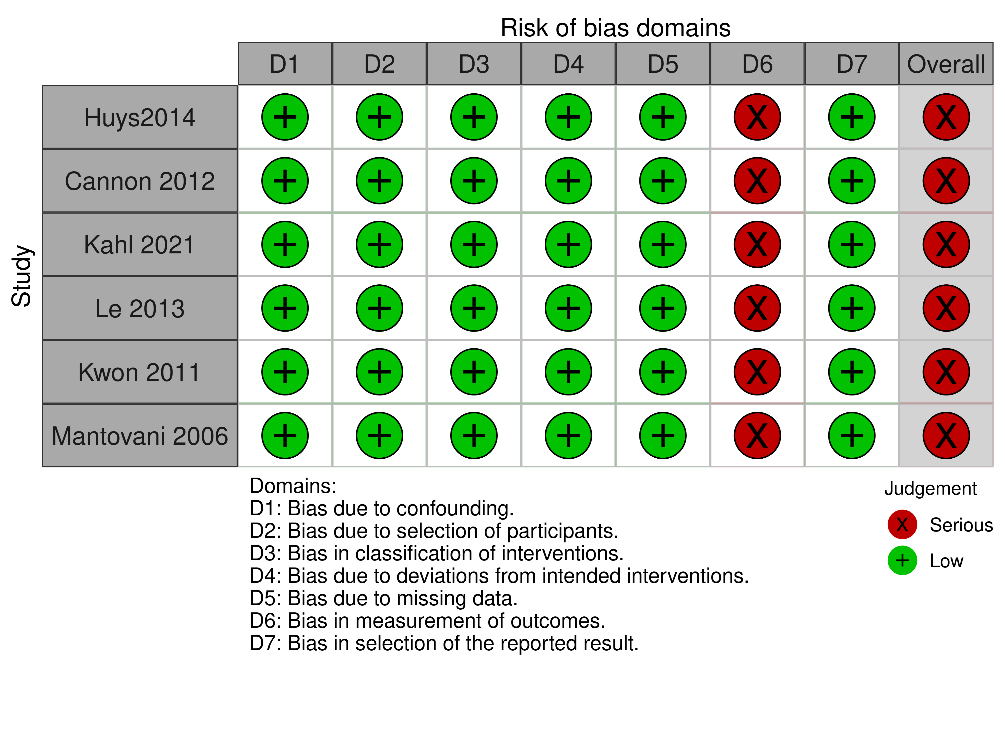


**Supplementary figure S3.** Sensitivity analysis by removing Kahl for YGTSS rTMS RCTs and non-RCTs


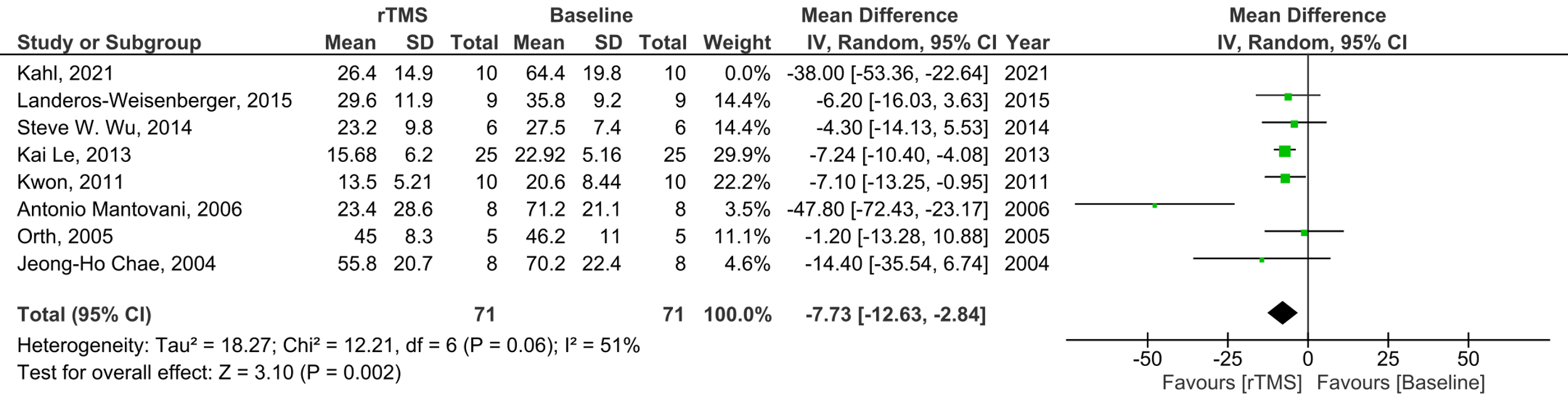


**Supplementary figure S4.** Sensitivity analysis by removing cannon for YGTSS DBS target area Gpi


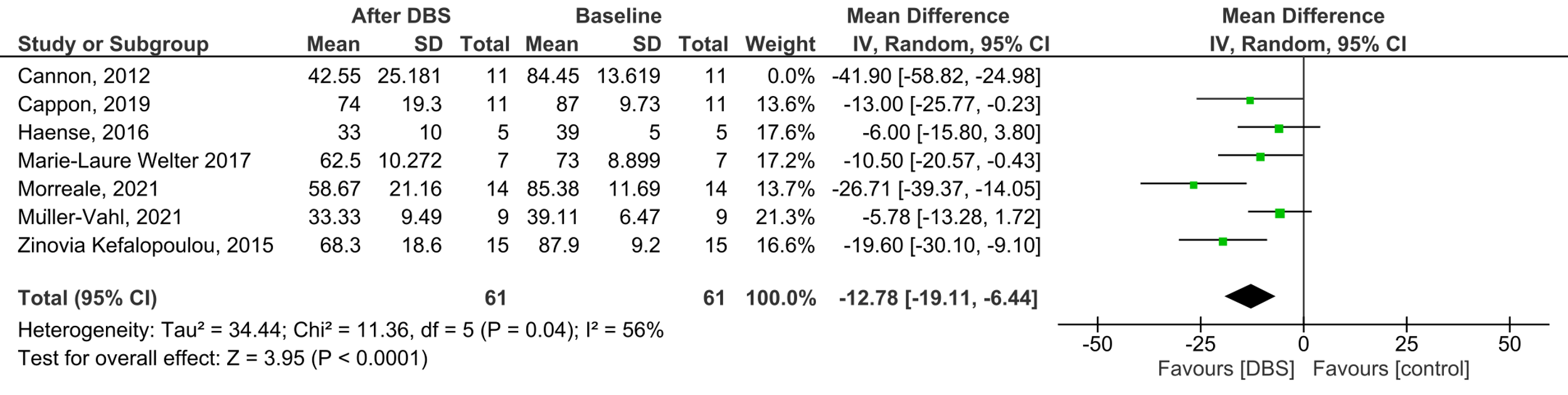


**Supplementary figure S5.** Sensitivity analysis by removing Haensae for YGTSS DBS target area ventrooral


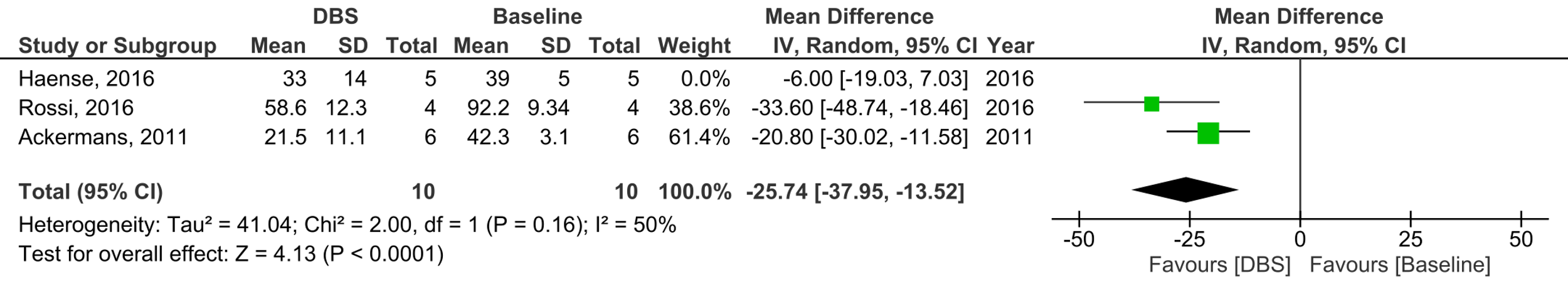


**Supplementary figure S6.** Publication Bias YGTSS


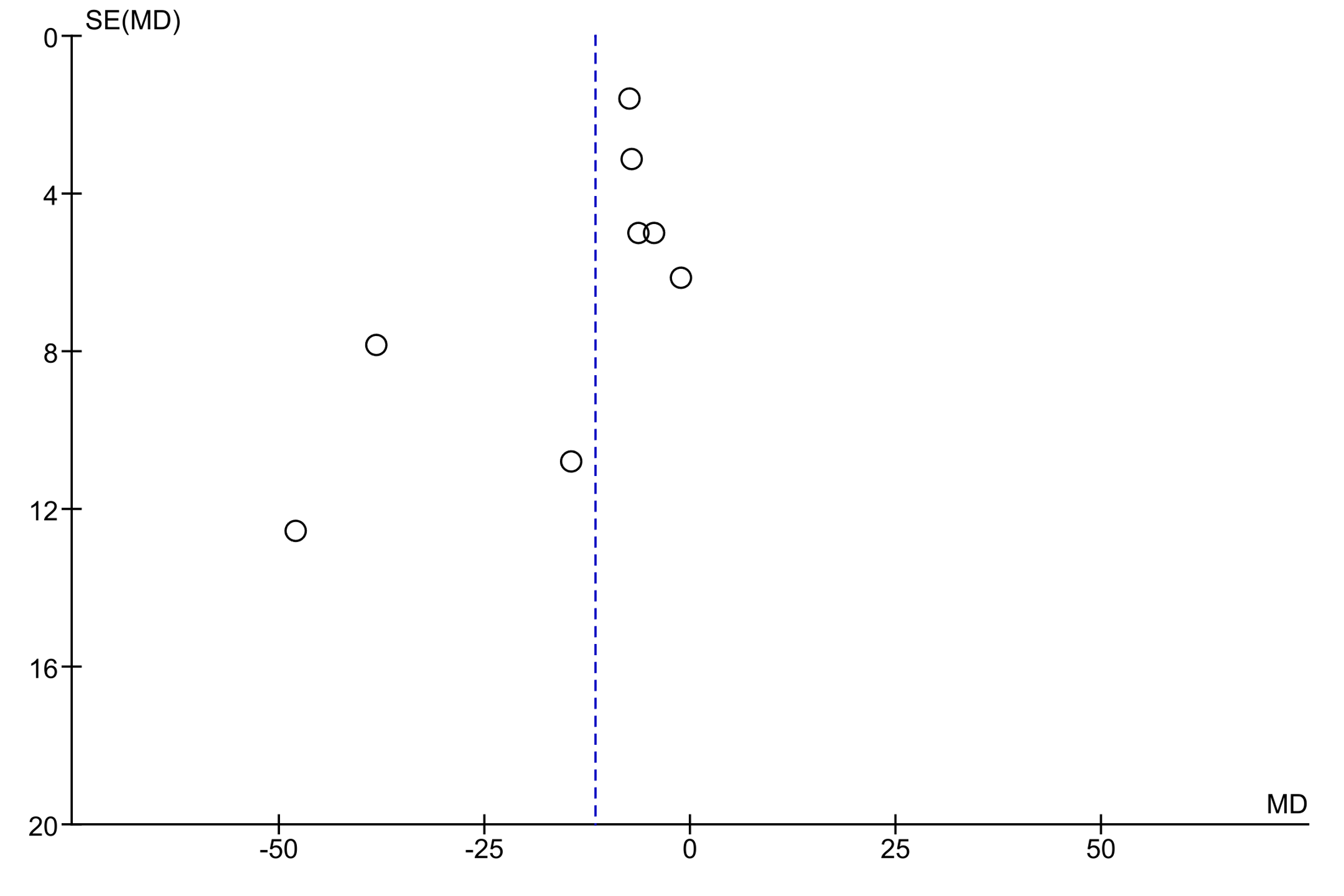


B- rTMS RCTs and non-RCTs


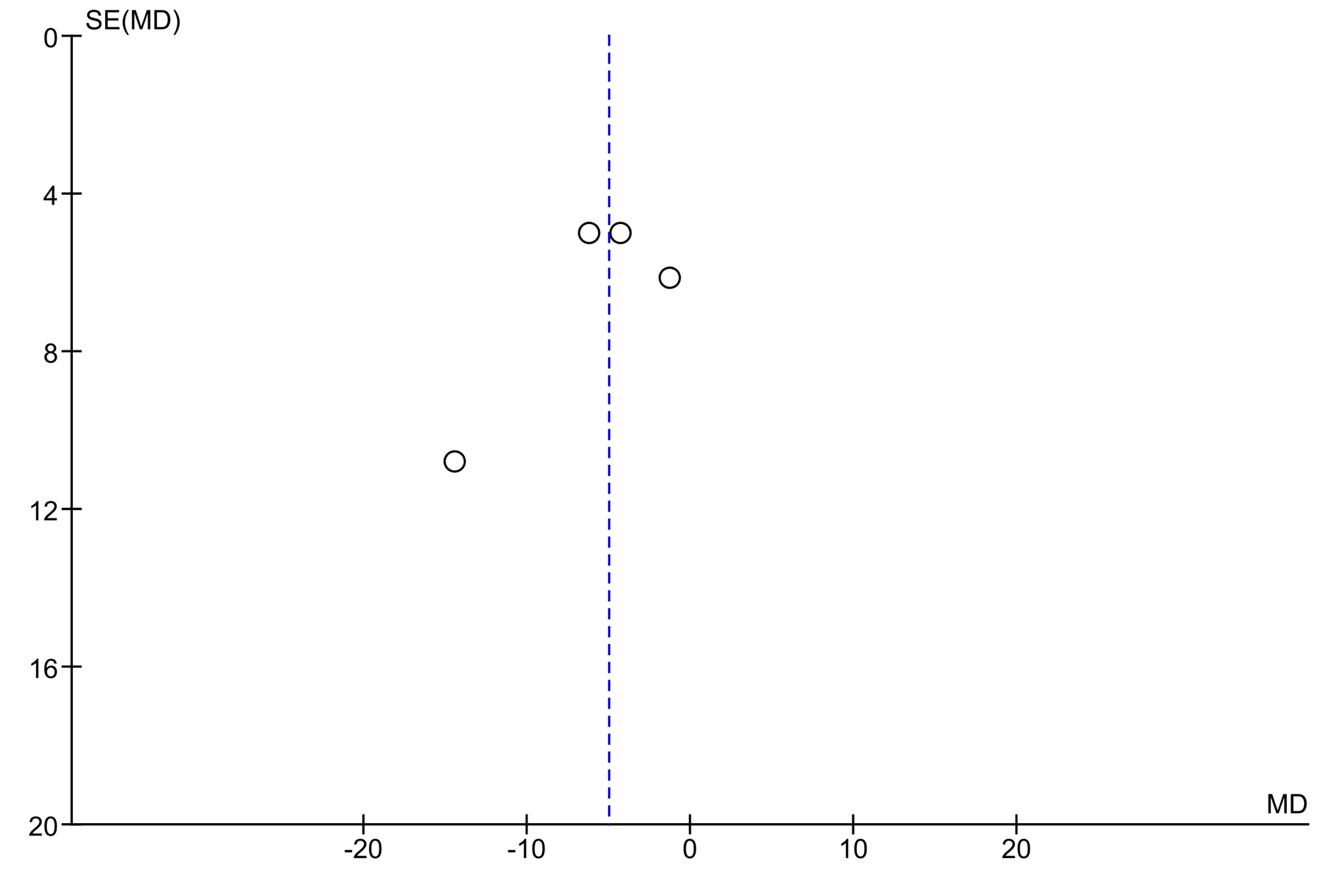


A- rTMS RCTs

C- rTMS SHAM


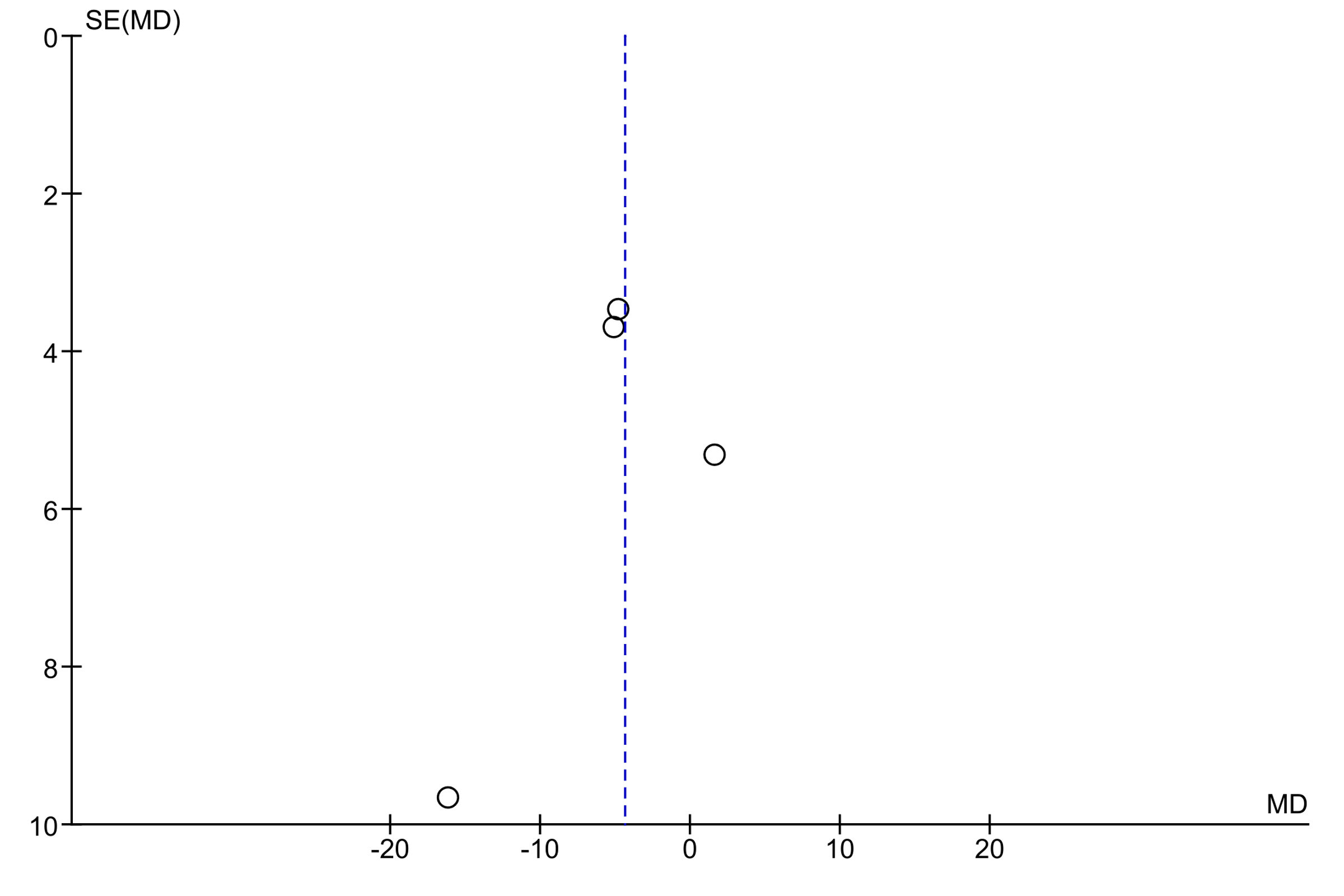


F- DBS SHAM


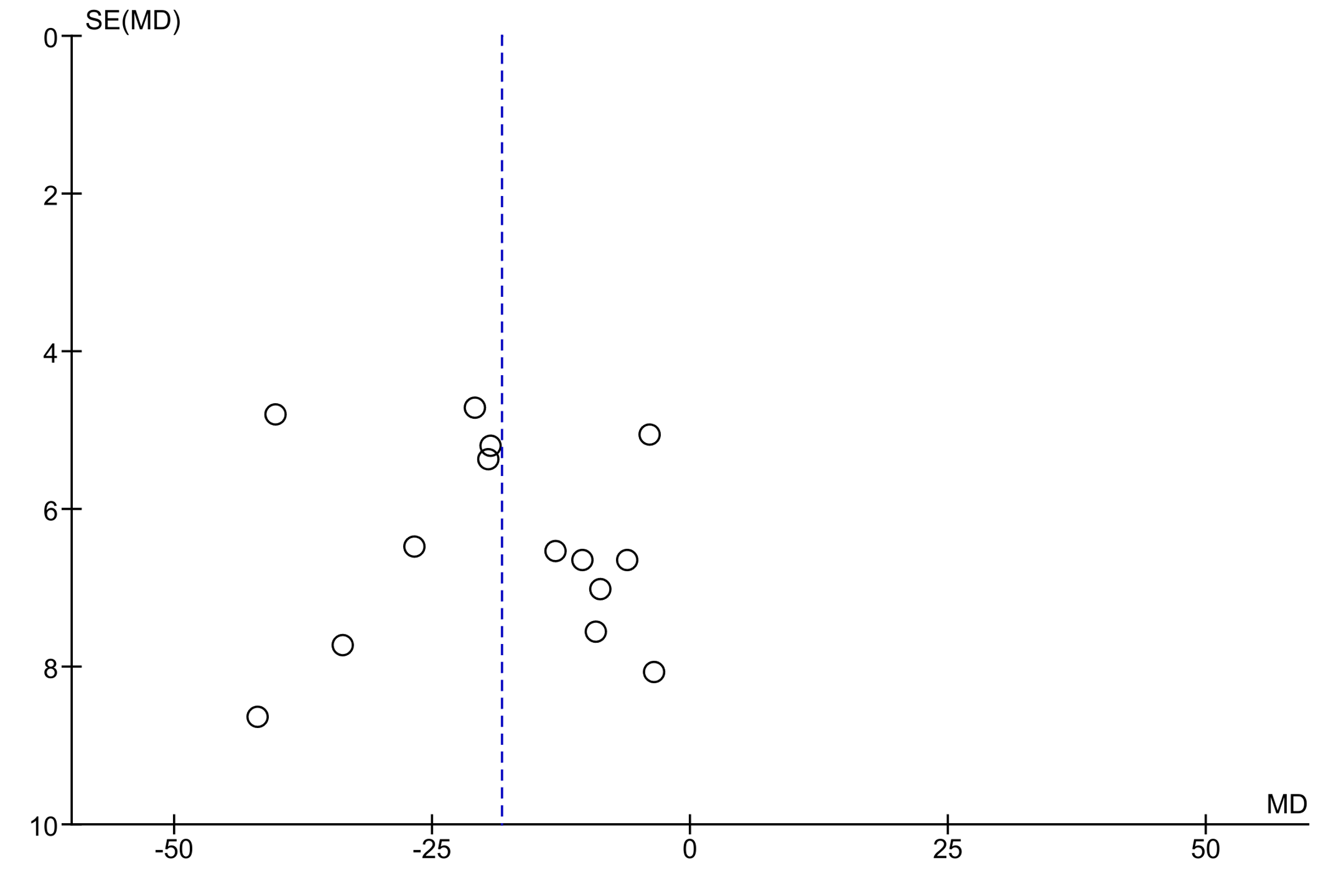


E- DBS RCTs and non-RCTs


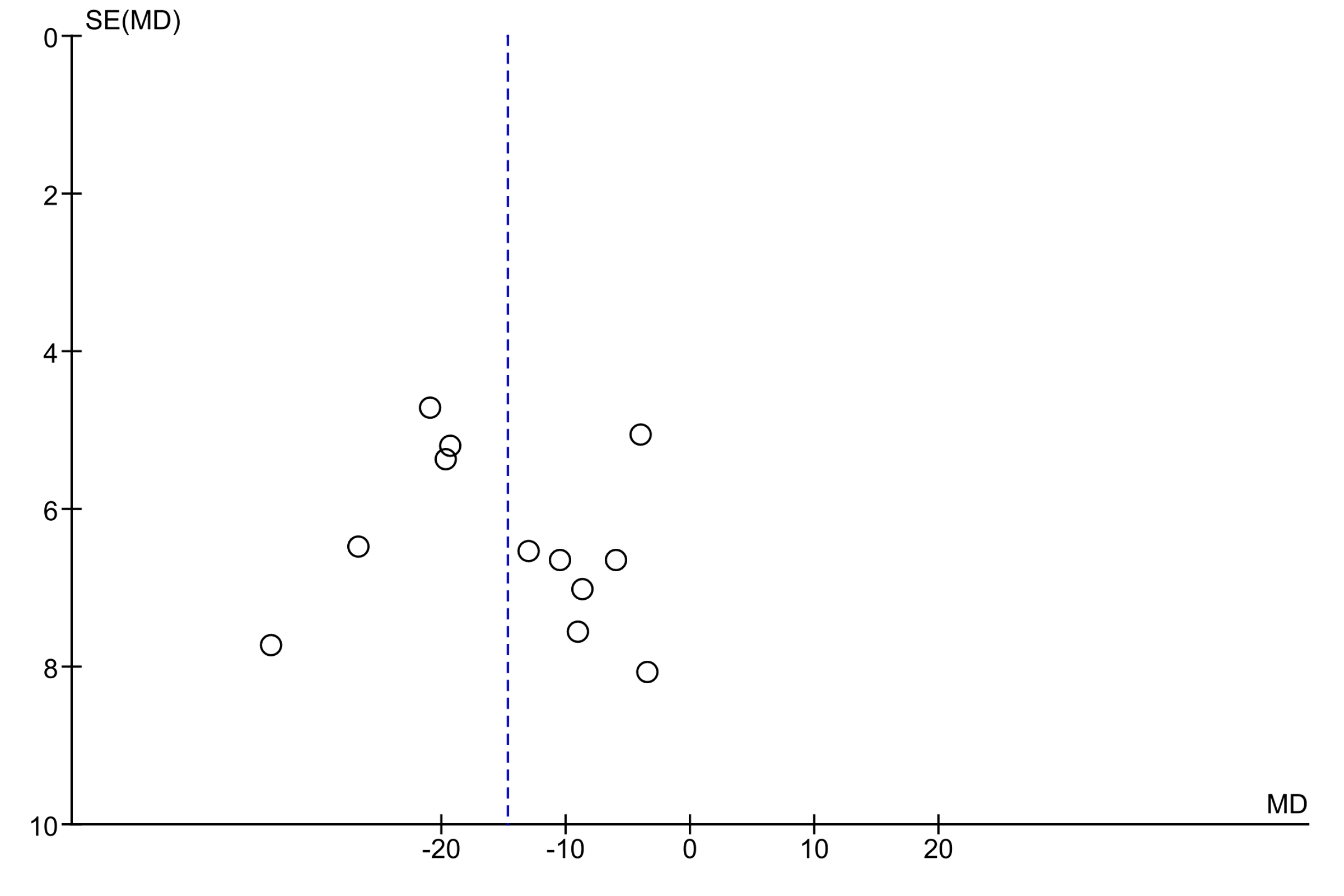


D- DBS RCTs


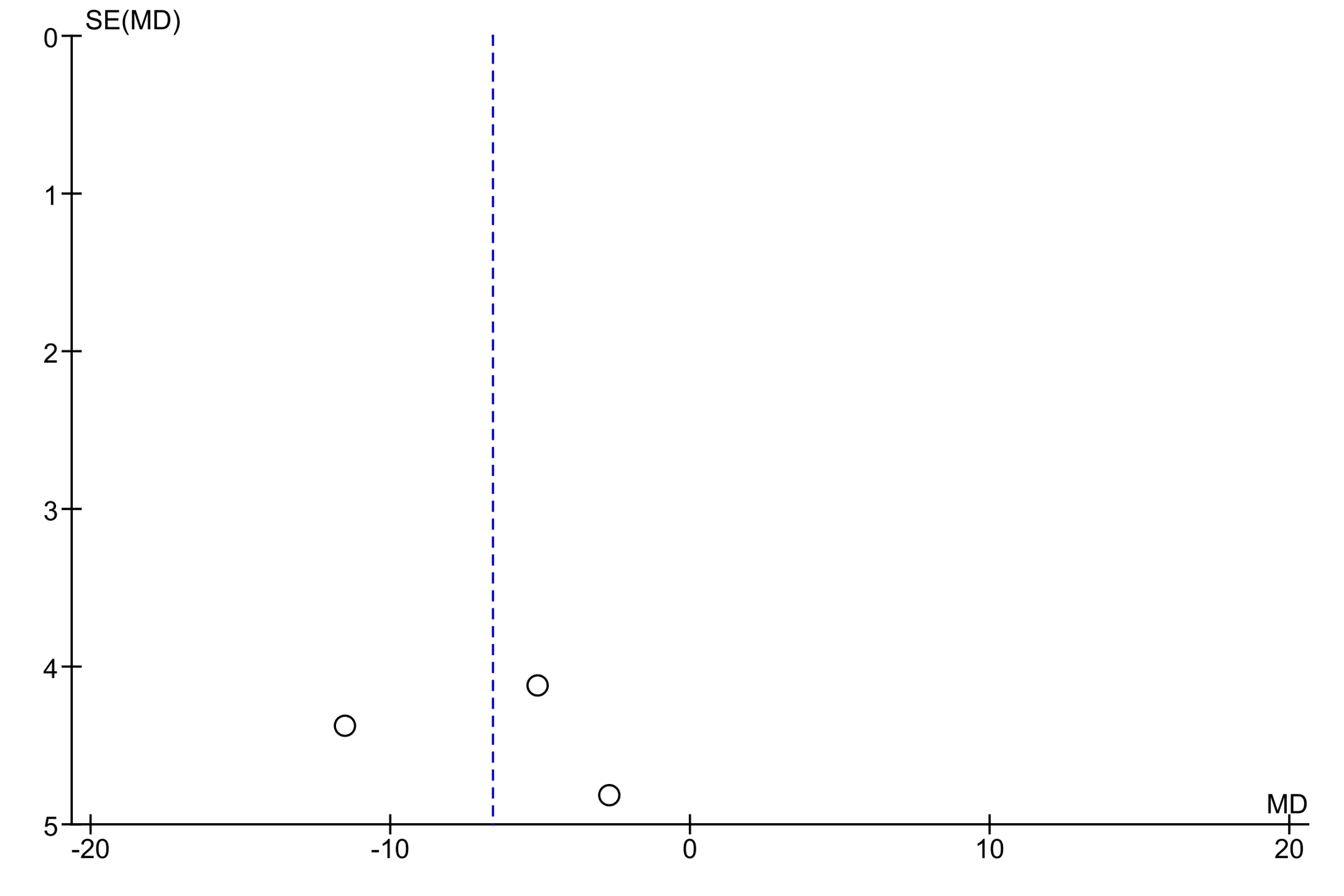


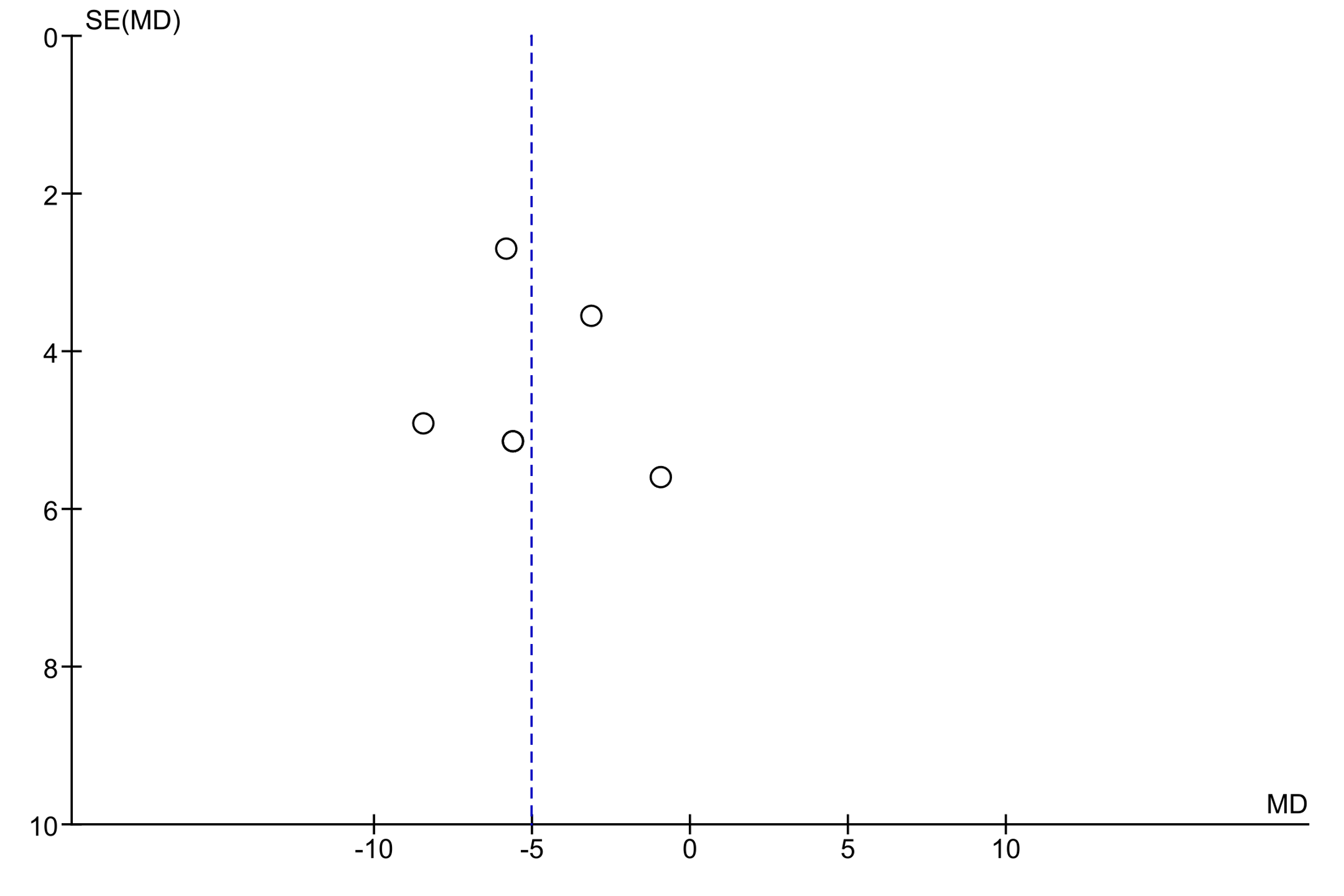


C- rTMS SHAM


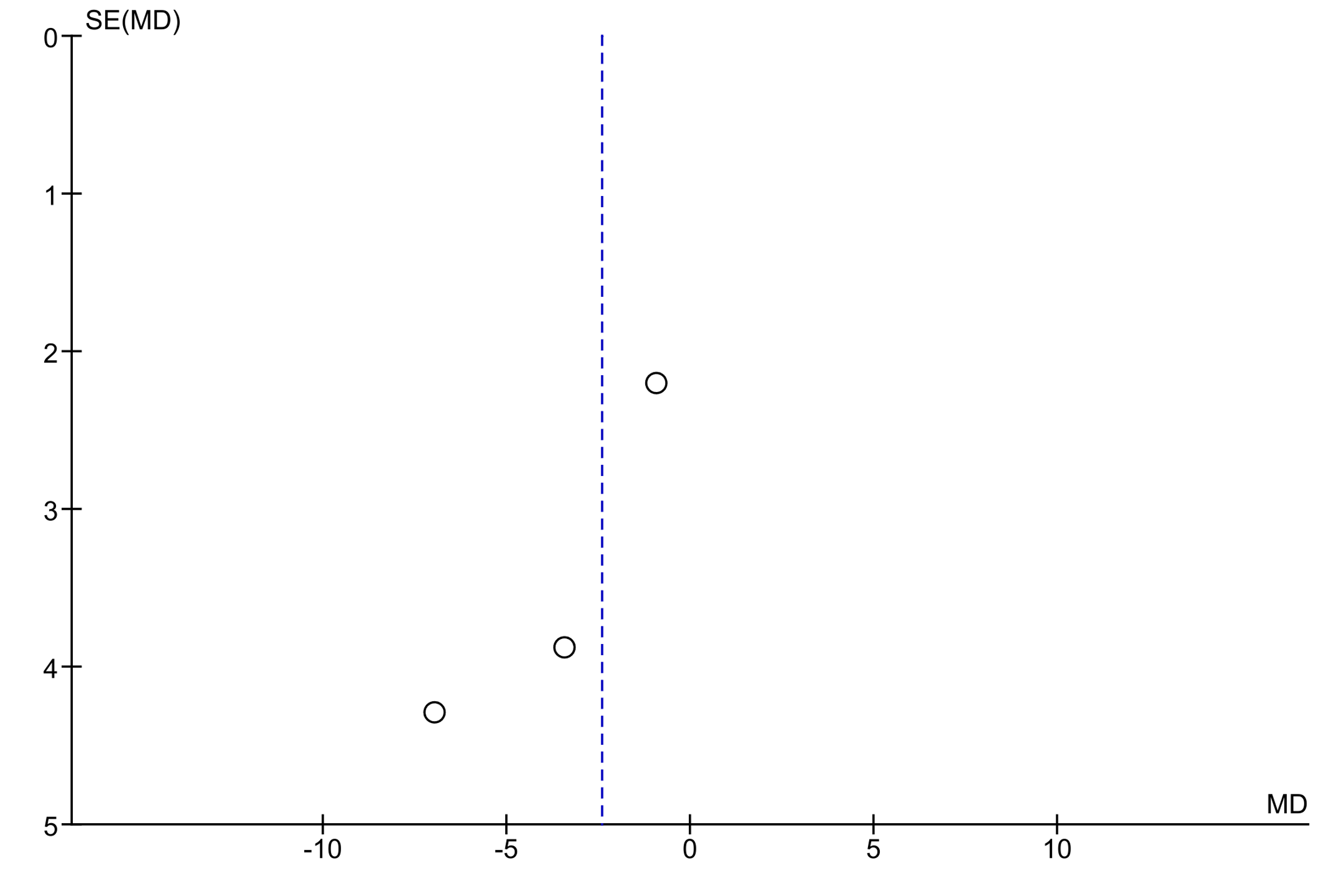


B- rTMS RCTs and non-RCTs


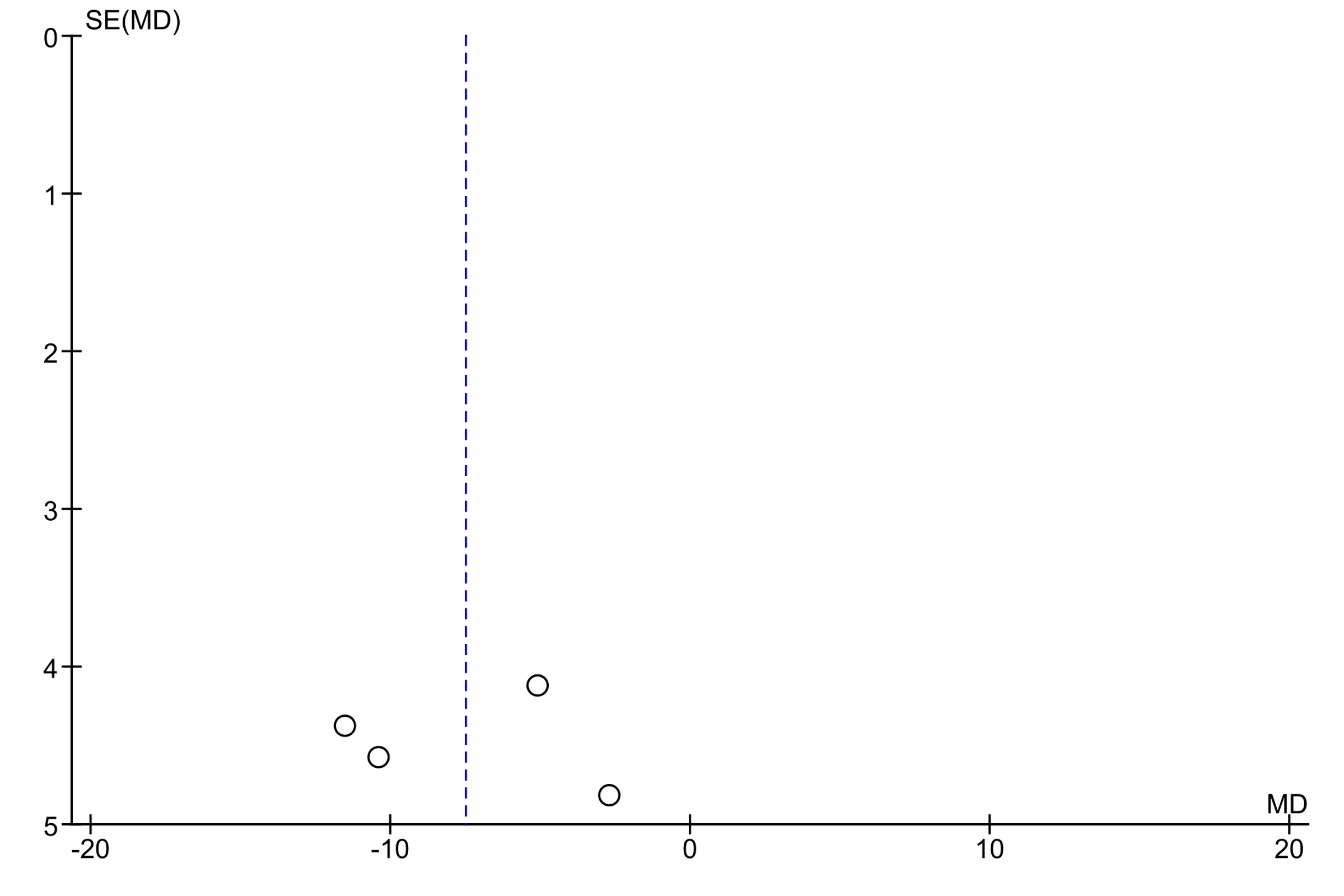


A- rTMS RCTs

**Supplementary figure S7.** Publication Bias YBOCS


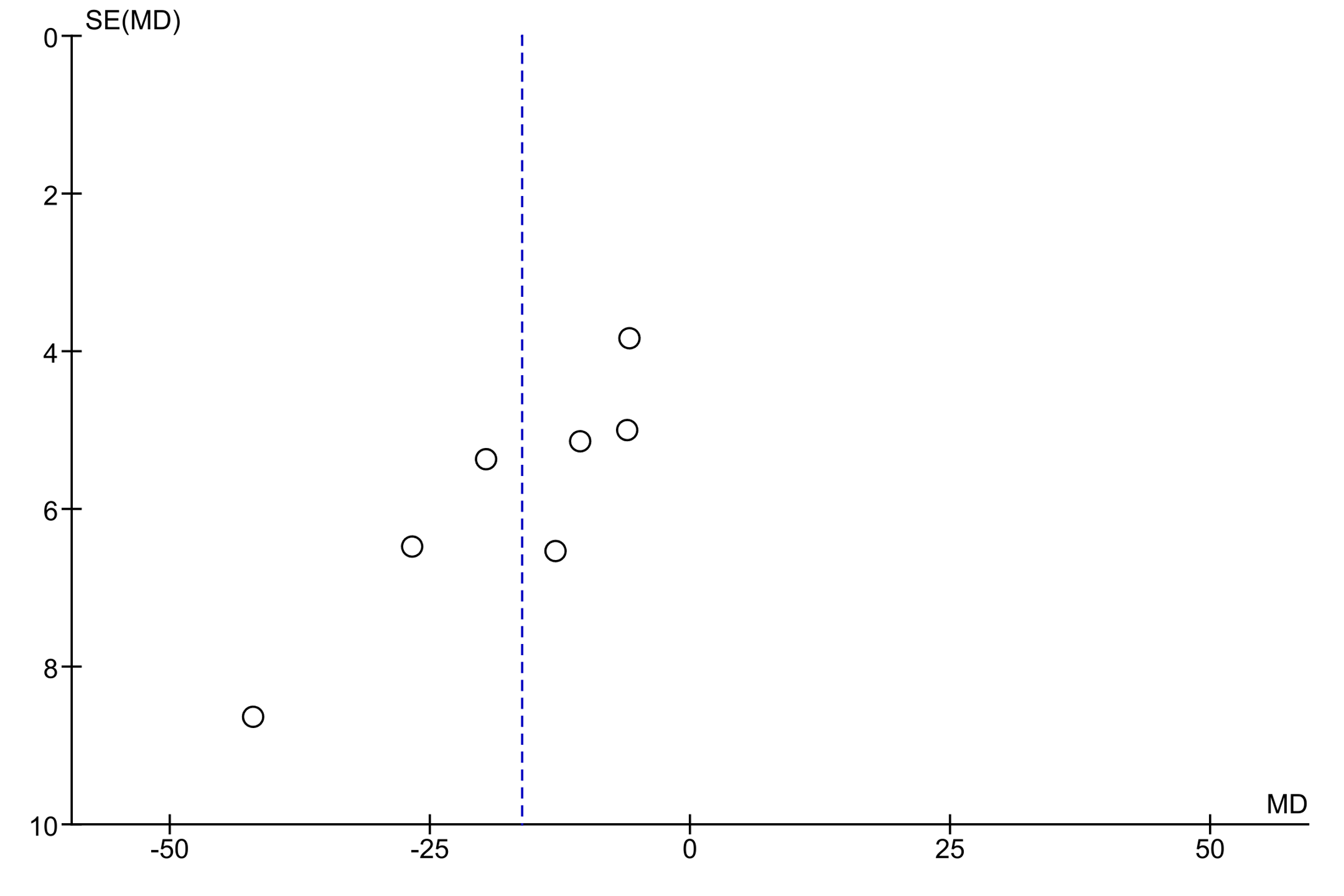


F- DBS SHAM


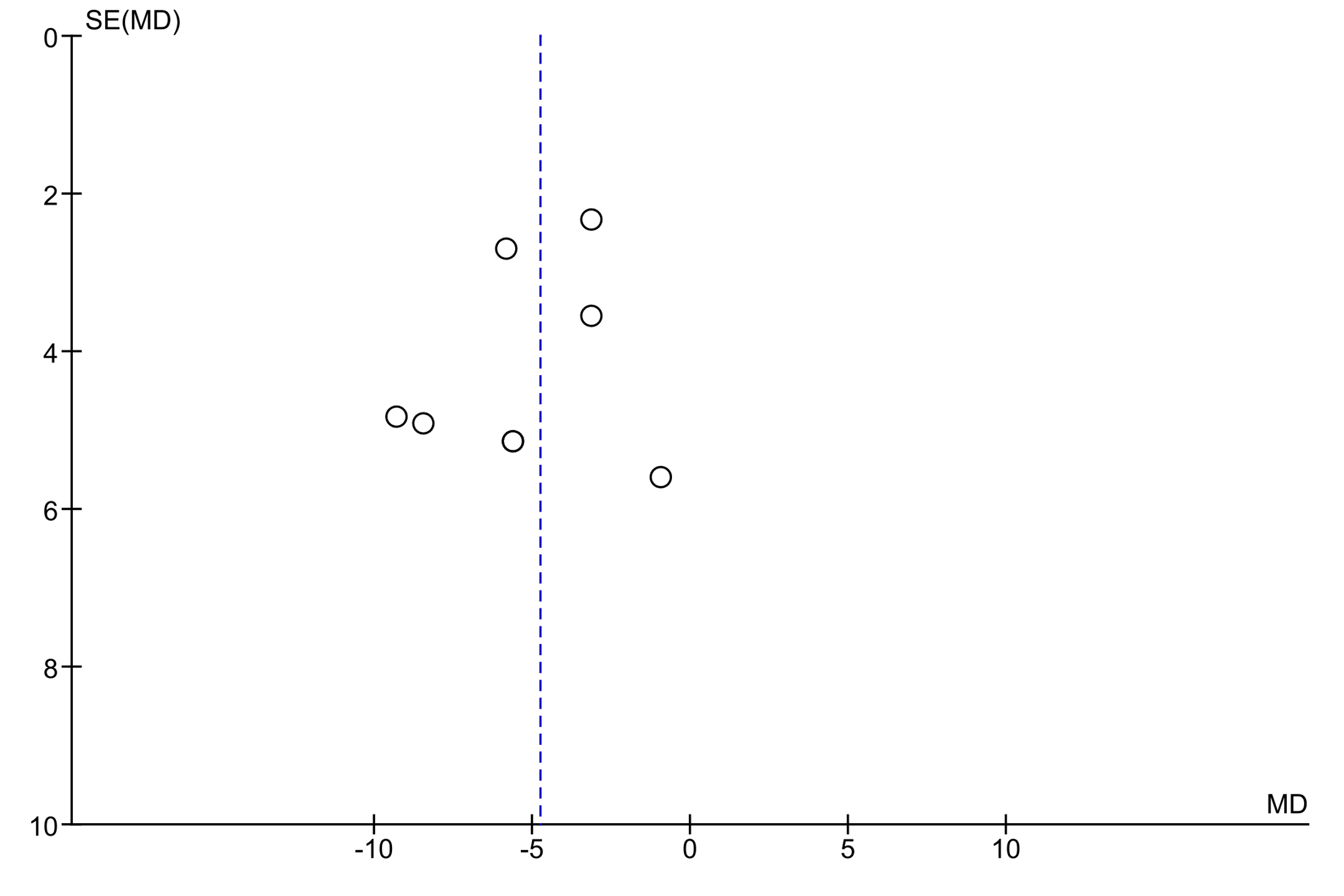


D- DBS RCTs


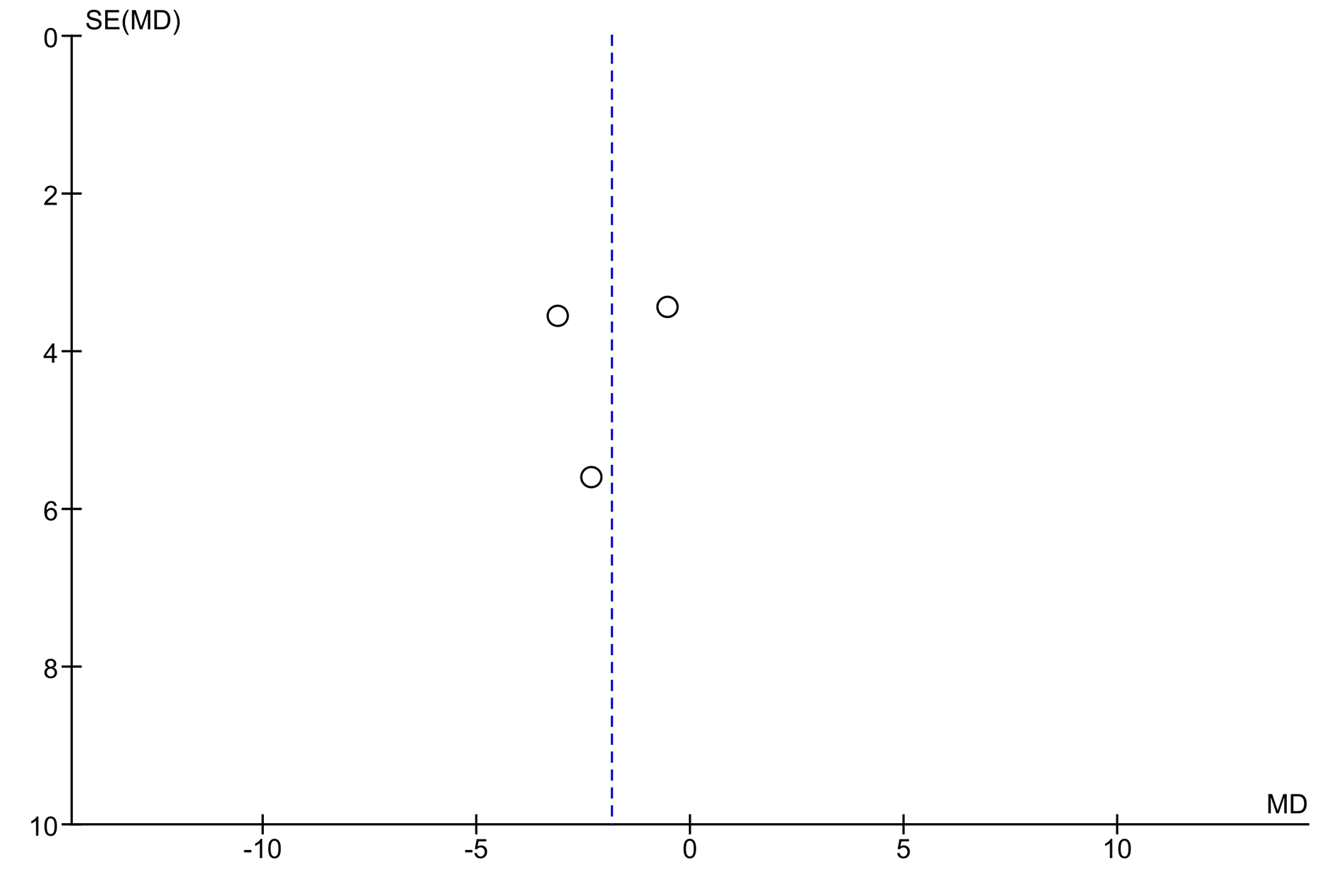


E- DBS RCTs and non-RCTs

**Supplementary figure S8.** Publication bias by target area

B- YGTSS, DBS, Thalamic ventro-oral

A- YGTSS, DBS, GPi


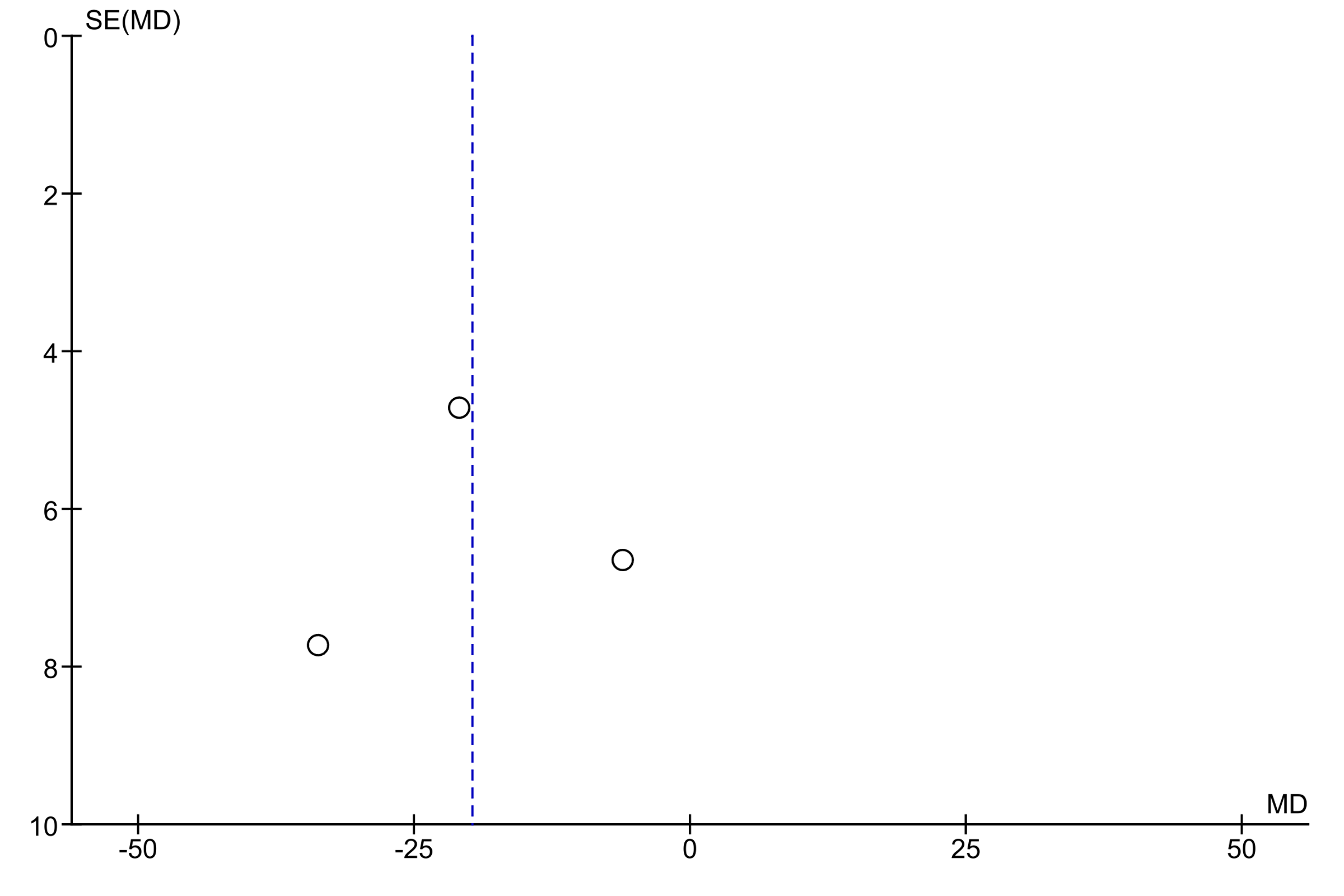

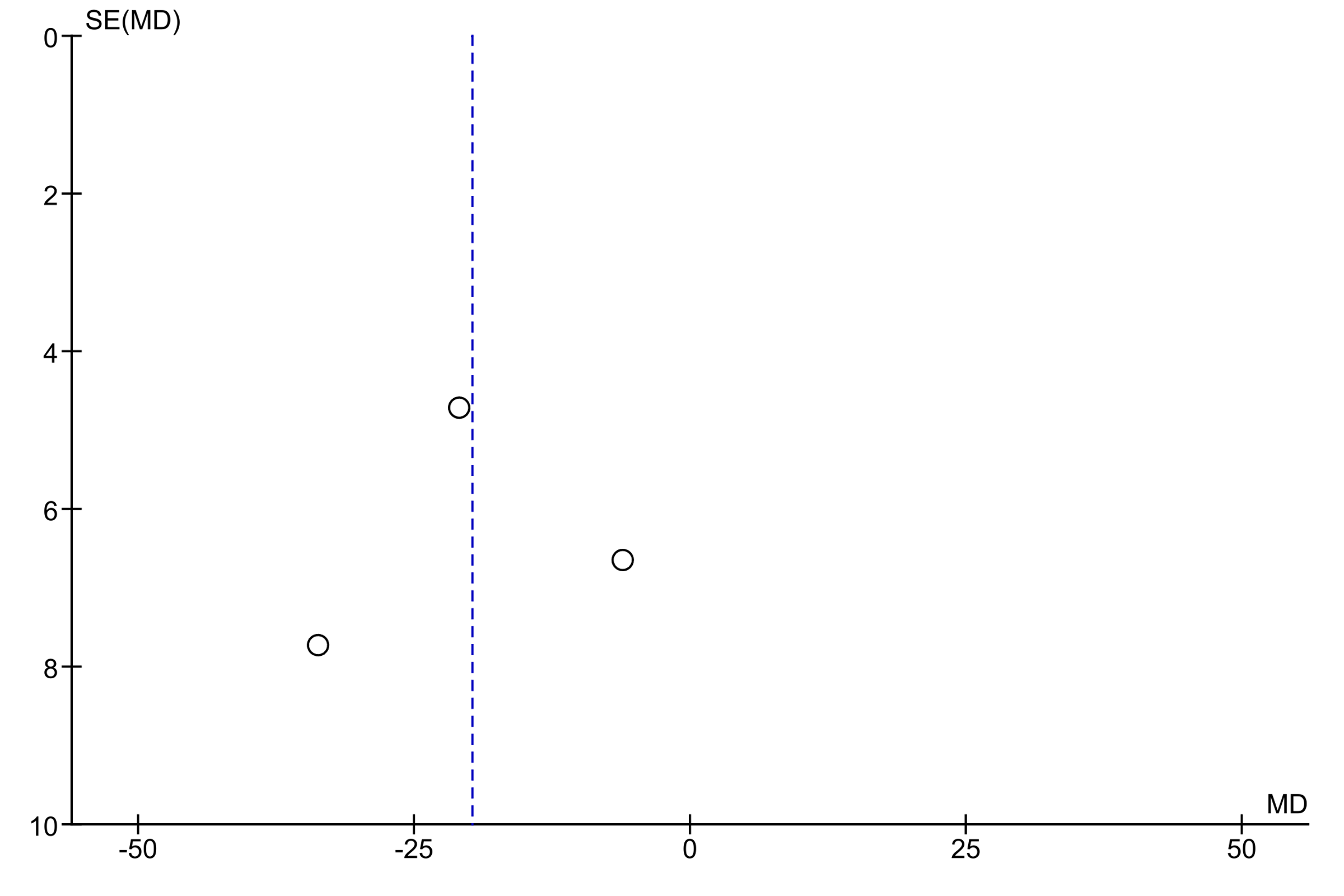


**Supplementary Table S2**. DBS studies side effects summary

| Article and year | Infection | Increase in tic severity | Paranesthesia | Mood deterioration | Hardware malfunction | Increase in anxiety | Visual disturbances | Weight gain | Sleep disturbances | Dysarthria | Headache | Dizziness | Tremor | Tiredness/lack of energy | Irritability | Depressive signs |
| --- | --- | --- | --- | --- | --- | --- | --- | --- | --- | --- | --- | --- | --- | --- | --- | --- |
| Morreale et al. 2021 | NR | NR | NR | NR | NR | NR | NR | NR | NR | NR | NR | NR | NR | NR | NR | NR |
| Baldermann et al. 2021 | NR | 2 | NR | NR | 1 | NR | NR | NR | NR | 1 | NR | 1 | NR | NR | 2 | NR |
| Müller-Vahl et al. 2021 | 2 | 1 | 2 | NR | 1 | NR | NR | NR | 1 | NR | 2 | 3 | 1 | 2 | NR | NR |
| Cappon et al. 2019 | NR | NR | NR | NR | NR | NR | NR | NR | NR | NR | NR | NR | NR | NR | NR | NR |
| Welter et al. 2017 | 5 | 17 | NR | NR | NR | NR | NR | 2 | 2 | 3 | 5 | NR | NR | 1 | NR | 4 |
| Haense et al. 2016 | NR | NR | NR | NR | NR | NR | NR | NR | NR | NR | NR | NR | NR | NR | NR | NR |
| Rossi et al. 2016 | NR | NR | NR | NR | NR | NR | NR | NR | NR | NR | NR | NR | NR | NR | NR | NR |
| Schoenberg et al. 2015 | NR | NR | NR | NR | NR | NR | NR | NR | NR | NR | NR | NR | NR | NR | NR | NR |
| Kefalopoulou et al. 2015 | 4 | NR | NR | 1 | NR | 2 | NR | NR | NR | 1 | 2 | NR | NR | 2 | 3 | NR |
| Huys et al. 2014 | 1 | 2 | 1 | 6 | NR | NR | NR | 2 | 2 | 6 | 1 | NR | 2 | NR | NR | NR |
| Cannon et al. 2012 | 1 | 1 | NR | NR | 3 | 2 | NR | NR | NR | NR | NR | NR | NR | NR | NR | NR |
| Ackermans et al. 2011 | 1 | NR | NR | NR | NR | NR | 6 | NR | NR | NR | NR | NR | NR | 7 | NR | NR |
| MACIUNRS et al. 2007 | NR | NR | NR | NR | NR | NR | NR | NR | NR | NR | NR | NR | NR | NR | NR | NR |
|  | 14 | 23 | 3 | 7 | 5 | 4 | 6 | 4 | 5 | 11 | 10 | 4 | 3 | 12 | 5 | 4 |

NR = Not reported

**Supplementary Table S3**. rTMS studies side effects summary

| Article and year | Headache | Scalp pain | Increased excitability | Abdominal pain | Sleepiness | Neck ache | Dry eyes |
| --- | --- | --- | --- | --- | --- | --- | --- |
| Kahl et al. 2021 | NR | NR | NR | NR | NR | NR | NR |
| Landero-Weinberger et al. 2015 | 5 | 1 | NR | NR | NR | 1 | NR |
| Wu et al. 2013 | 3 | NR | NR | 3 | NR | NR | 3 |
| Le et al. 2013 | NR | NR | NR | NR | 1 | NR | NR |
| Kwon et al. 2011 | NR | 1 | NR | NR | NR | NR | NR |
| Mantovani et al. 2006 | NR | NR | NR | NR | NR | NR | NR |
| Orth et al. 2005 | NR | NR | NR | NR | NR | NR | NR |
| Chae et al. 2004 | 3 | NR | 1 | NR | NR | NR | NR |
|  | 11 | 2 | 1 | 3 | 1 | 1 | 3 |

NR = Not reported
